# Supplementary material for: Novel immunomodulatory properties of adenosine analogs promote their antiviral activity against SARS-CoV-2
Source: EMBO Rep. 2024 Jul 15;25(8):21. doi: 10.1038/s44319-024-00189-4 (PMC11315900; doi:10.1038/s44319-024-00189-4)
Supplement: Supplementary file 1 — Appendix [file 44319_2024_189_MOESM1_ESM.pdf]

Appendix Figures

Table of Contents

	Page
• Appendix Figure S1	1
• Appendix Figure S2	2
• Appendix Figure S3	3

Appendix Figure S1

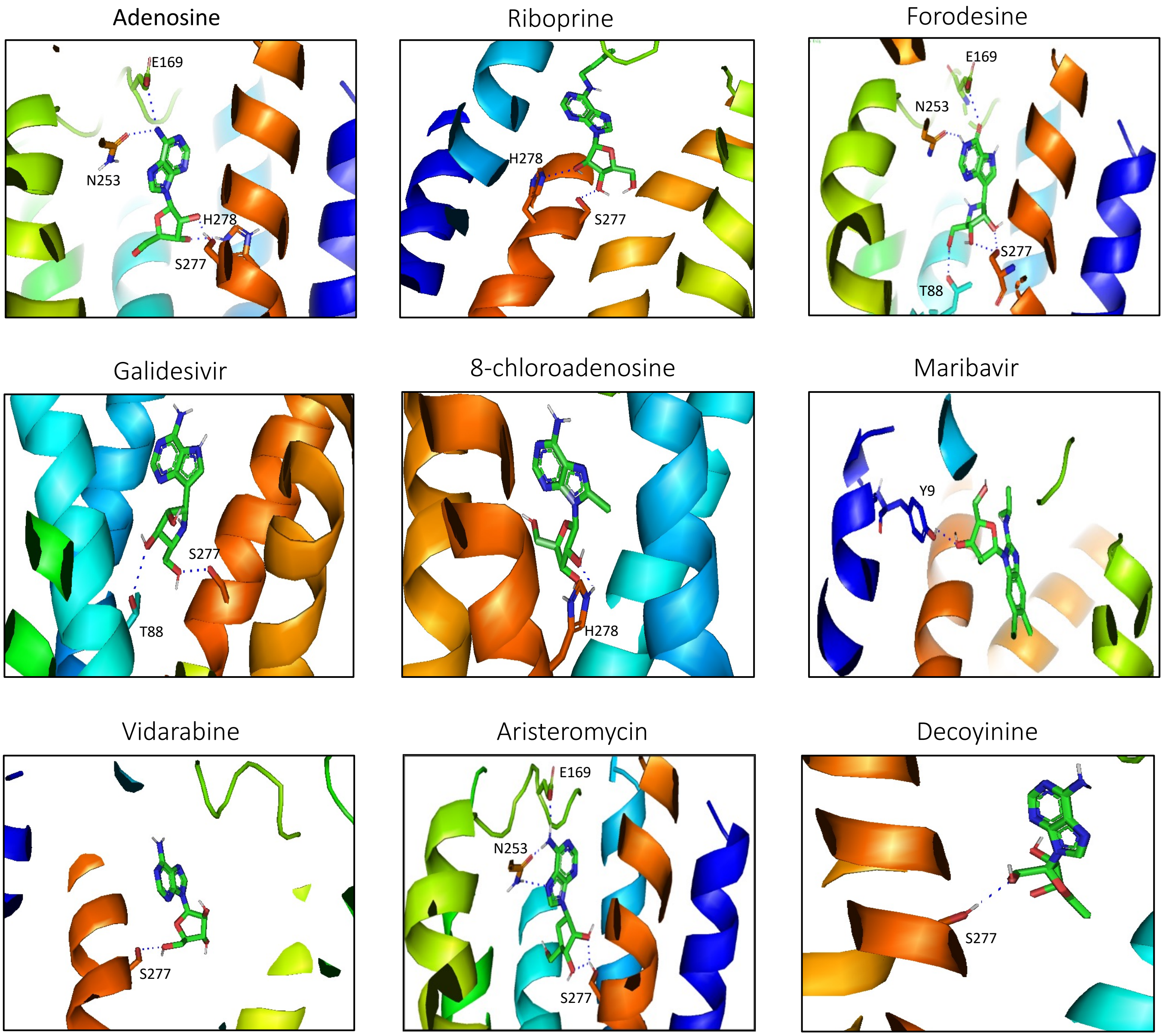

Appendix Figure S1 - Molecular docking of adenosine and adenosine analogs with A2AR. Computational modelling of adenosine, Riboprine (Pubchem CID: 24405), Forodesine (Pubchem CID: 135409409), Galidesivir (Pubchem CID: 10445549), 8-Chloroadenosine (Pubchem CID: 147569), Maribavir (Pubchem CID: 471161), Vidarabine (Pubchem CID: 21704), Aristeromycin (Pubchem CID: 65269), Decoyinine (Pubchem CID: 121578) and A2AR (PDB ID: 2YDO).

Appendix Figure S2

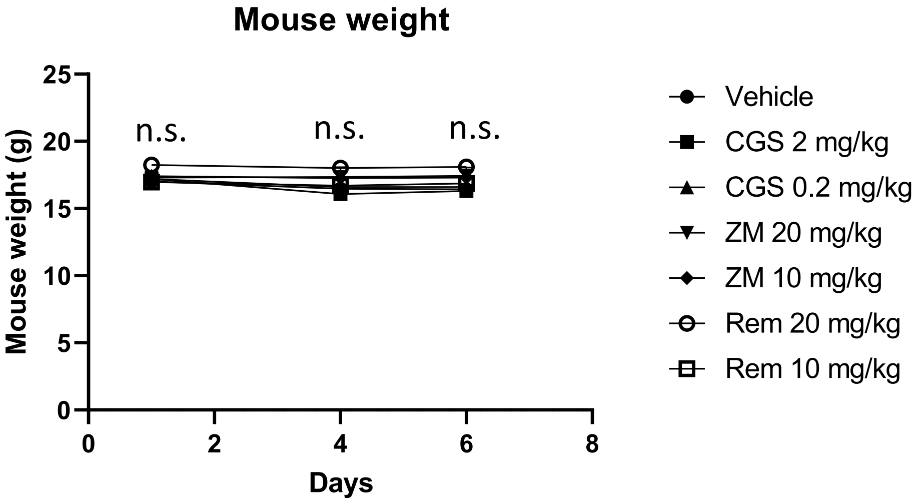

Appendix Figure S2 - Mouse weight change in response to A2AR engagement. Mouse weight change during 7-days treatment with different concentrations of CGS, ZM or REM in uninfected mice (n = 3 mice per group). Data information: the letter "n" indicates biological replicates. Data are presented as mean ± SD. Statistical significance was calculated by one-way ANOVA with Bonferroni correction. n.s. non-significant.

Appendix Figure S3

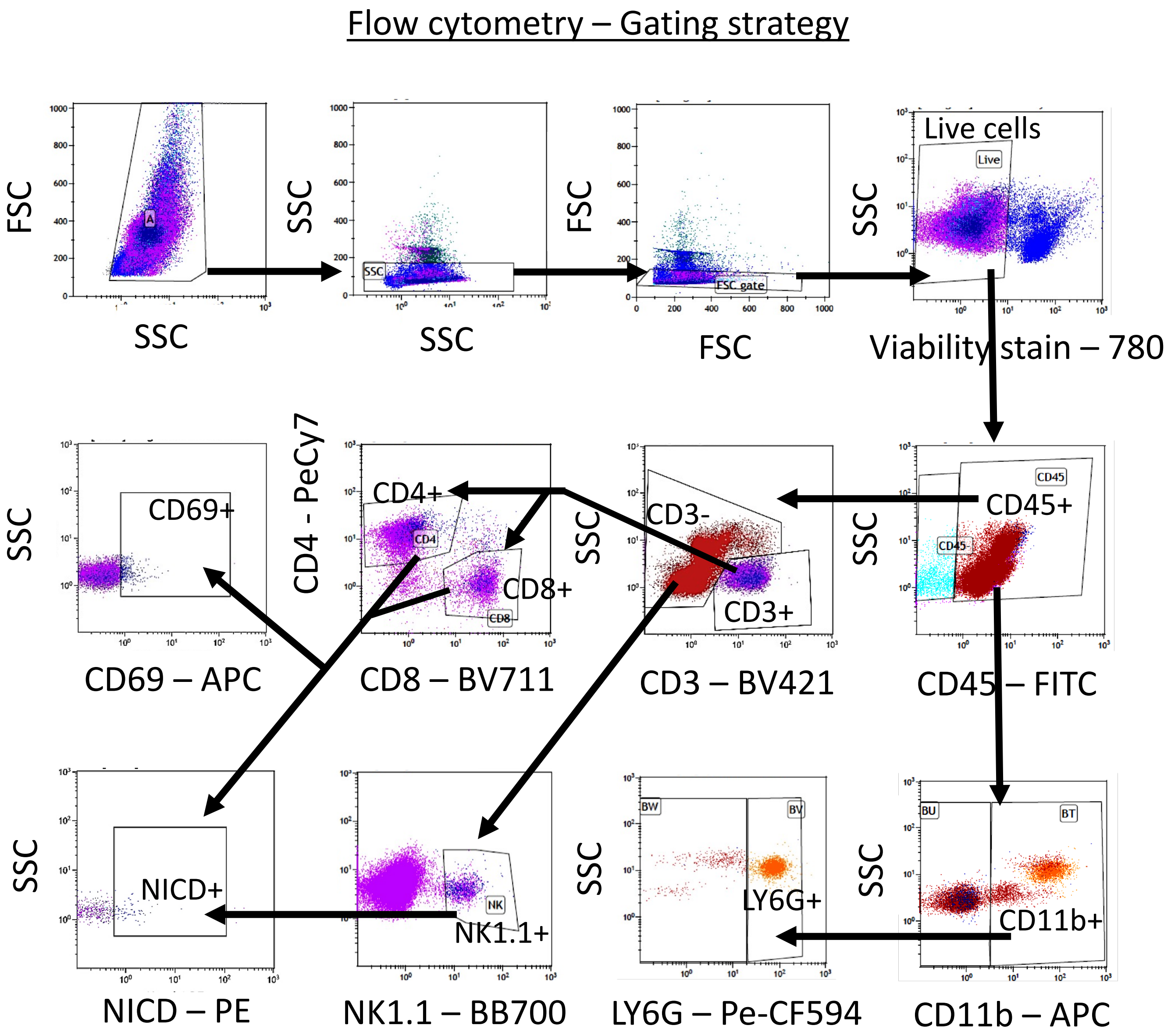

Appendix Figure S3 - Flow cytometry analysis. Gating strategy for cytofluorimetric analysis of mouse blood or spleen.
