# Supplementary material for: Novel immunomodulatory properties of adenosine analogs promote their antiviral activity against SARS-CoV-2
Source: EMBO Rep. 2024 Jul 15;25(8):21. doi: 10.1038/s44319-024-00189-4 (PMC11315900; doi:10.1038/s44319-024-00189-4)
Supplement: Supplementary file 8 — Expanded View Figures [file 44319_2024_189_MOESM8_ESM.pdf]

Expanded View Figures

Figure EV1. Structure similarities and molecular docking simulations between GS-441524 and adenosine.

(**A**) Crystallized structure (Top) and simulated structure (bottom) of adenosine binding to A2AR. (**B**) Structure of adenosine (PubChem ID 60961) (top) and GS (PubChem ID 44468216) (bottom). (**C**) Nine simulated states of GS binding to A2AR. Calculated binding affinities (ΔG, kcal/mol) are shown in the bottom table.

 © The Author(s)

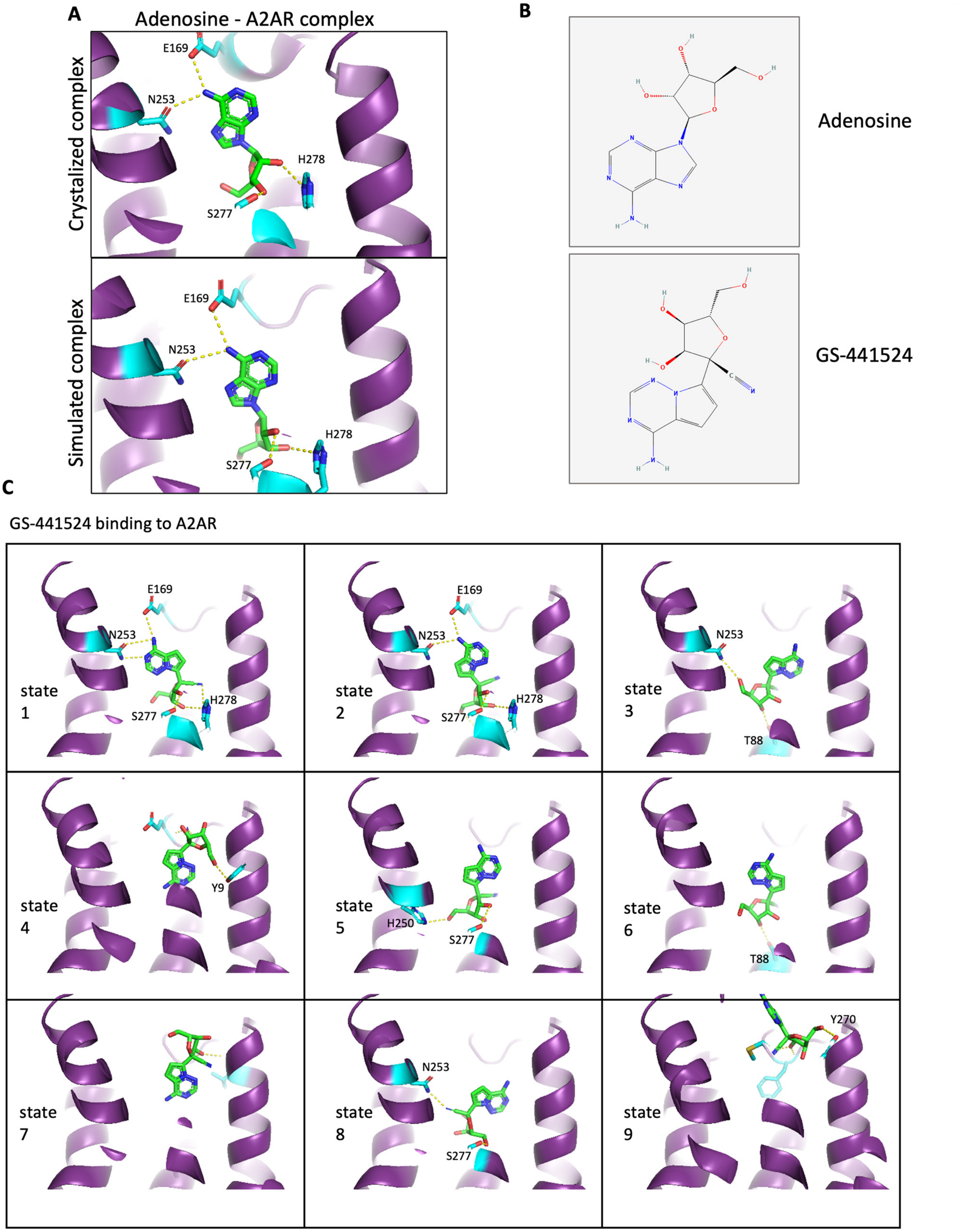

	Affinity for A2AR binding (ΔG)								
GS	-8.4	-8.3	-8.1	-8.1	-7.9	-7.8	-7.6	-7.5	-7.4

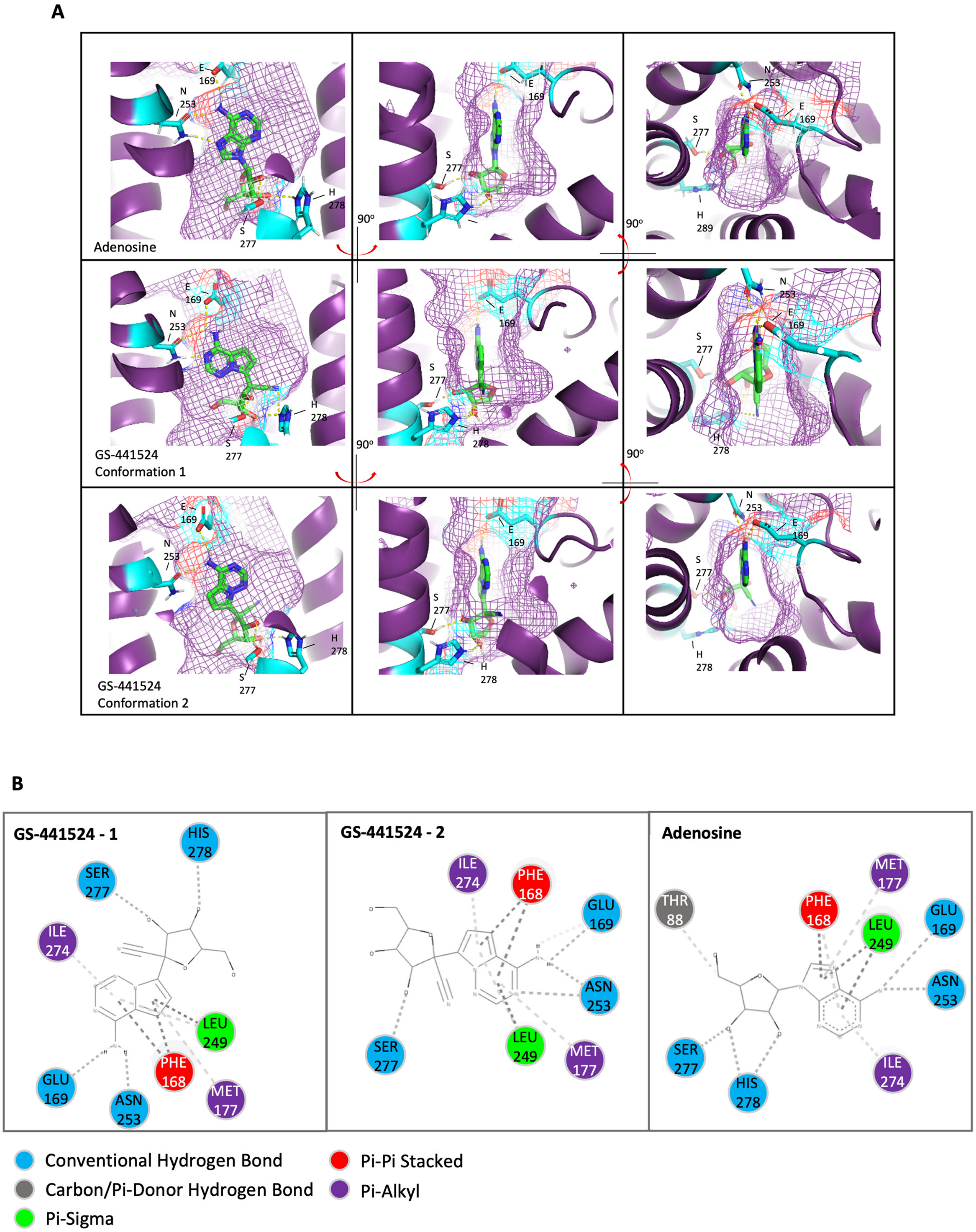
A
Adenosine
GS-441524
Conformation 1
GS-441524
Conformation 2
90°
B
GS-441524 - 1
GS-441524 - 2
Adenosine
Conventional Hydrogen Bond
Carbon/Pi-Donor Hydrogen Bond
Pi-Sigma
Pi-Pi Stacked
Pi-Alkyl

 © The Author(s)

Figure EV2. Detailed analysis of molecular bonds and interaction between GS-441524 and A2AR.

(**A**) Detailed presentation of Fig. 3D showing the surface (mesh) of E169, N253, S277, and H278 residues in the A2AR active site in contact with GS. (**B**) 2D presentation of detailed molecular bonds and interactions of GS confirmation 1 and 2 (left and middle panels) or adenosine (right panel) with A2AR predicted using Discovery Studio software.

© The Author(s)

A

GS-441524 binding to A1R

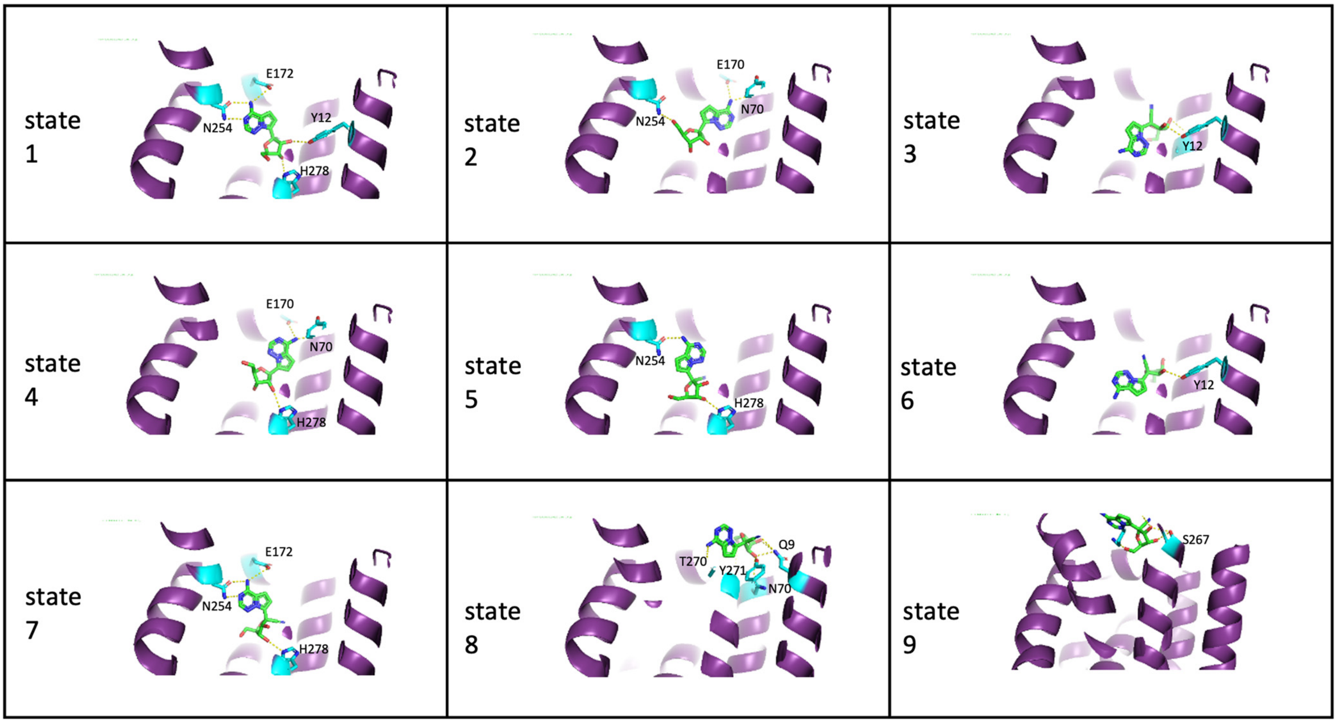

B

Adenosine binding to A1R

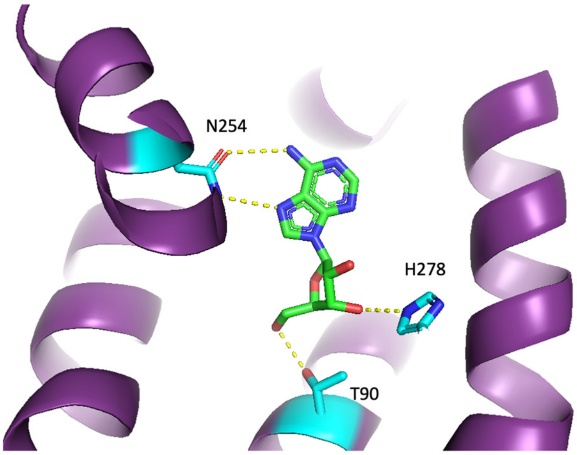

C

GS binding to A1R

Affinity for A1R binding (ΔG)		
mode	affinity (kcal/mol)	
	GS	adenosine
1	-6.8	-6.7
2	-6.6	-6.6
3	-6.2	-6.6
4	-5.6	-6.3
5	-5.4	-6.2
6	-5.3	-6.2
7	-5.2	-6.1
8	-5.2	-6.1
9	-5.2	-6.1

 © The Author(s)

Figure EV3. Molecular docking between GS-441524 and A1R.

(**A**) Simulated binding of GS to A1R (PDB ID: 6D9H). (**B**) Simulated binding of adenosine to A1R. (**C**) Calculated binding affinities (ΔG, kcal/mol) of GS and adenosine to A1R.

© The Author(s)

A

GS-441524 binding to A2BR

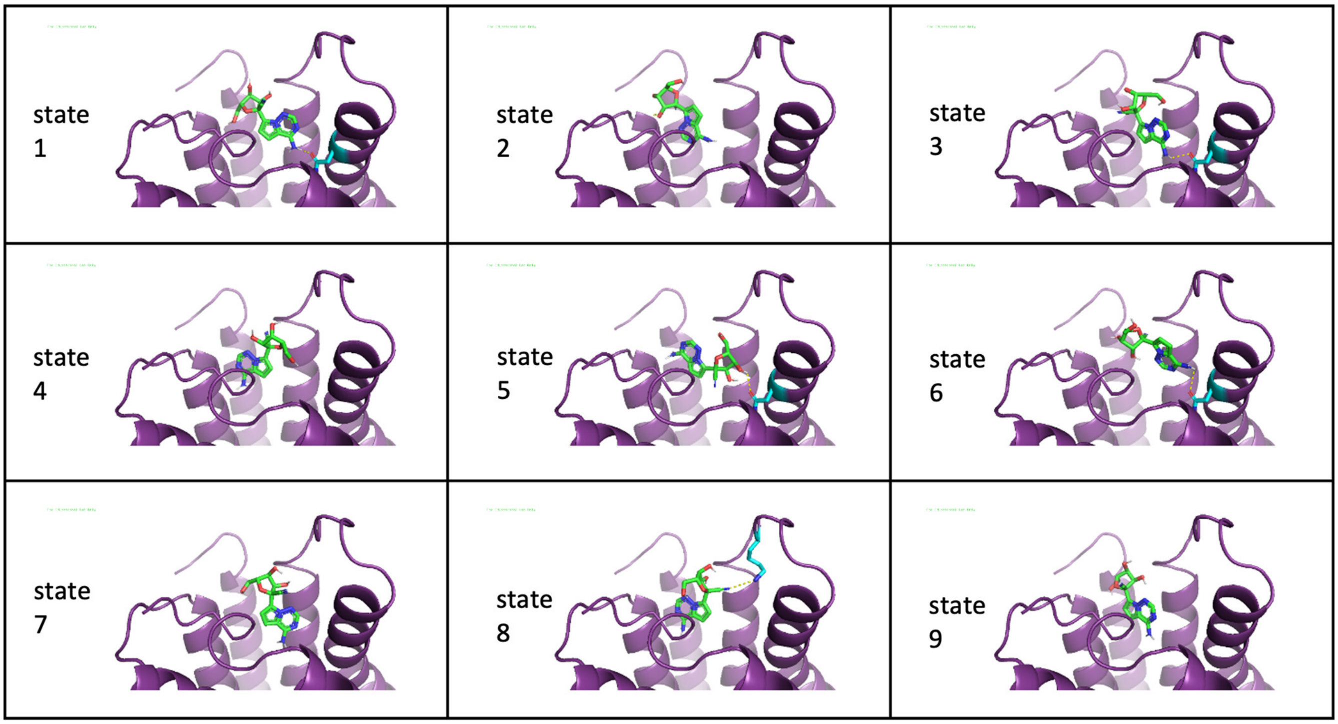

B

Adenosine binding to A2BR

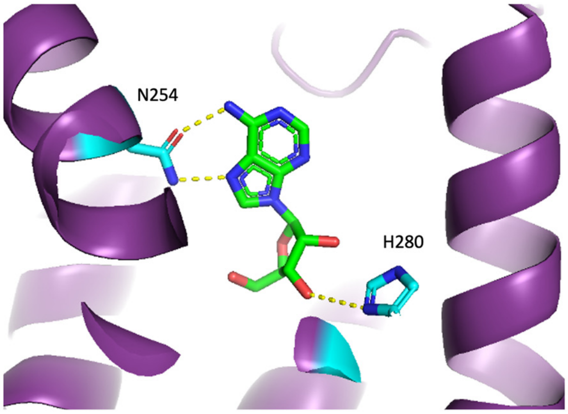

C

GS binding to A2BR

Affinity for A2BR binding (ΔG)		
mode	affinity (kcal/mol)	
	GS	adenosine
1	-5.6	-6.1
2	-5.5	-6
3	-5.3	-5.8
4	-5.2	-5.7
5	-5.2	-5.7
6	-5.1	-5.7
7	-5.1	-5.6
8	-5.1	-5.5
9	-5	-5.5

 © The Author(s)

Figure EV4. Molecular docking between GS-441524 and A2BR.

(**A**) Simulated binding of GS to A2BR (PDB ID: 8HDP). (**B**) Simulated binding of adenosine to A2BR. (**C**) Calculated binding affinities (ΔG, kcal/mol) of GS and adenosine to A2BR.

© The Author(s)

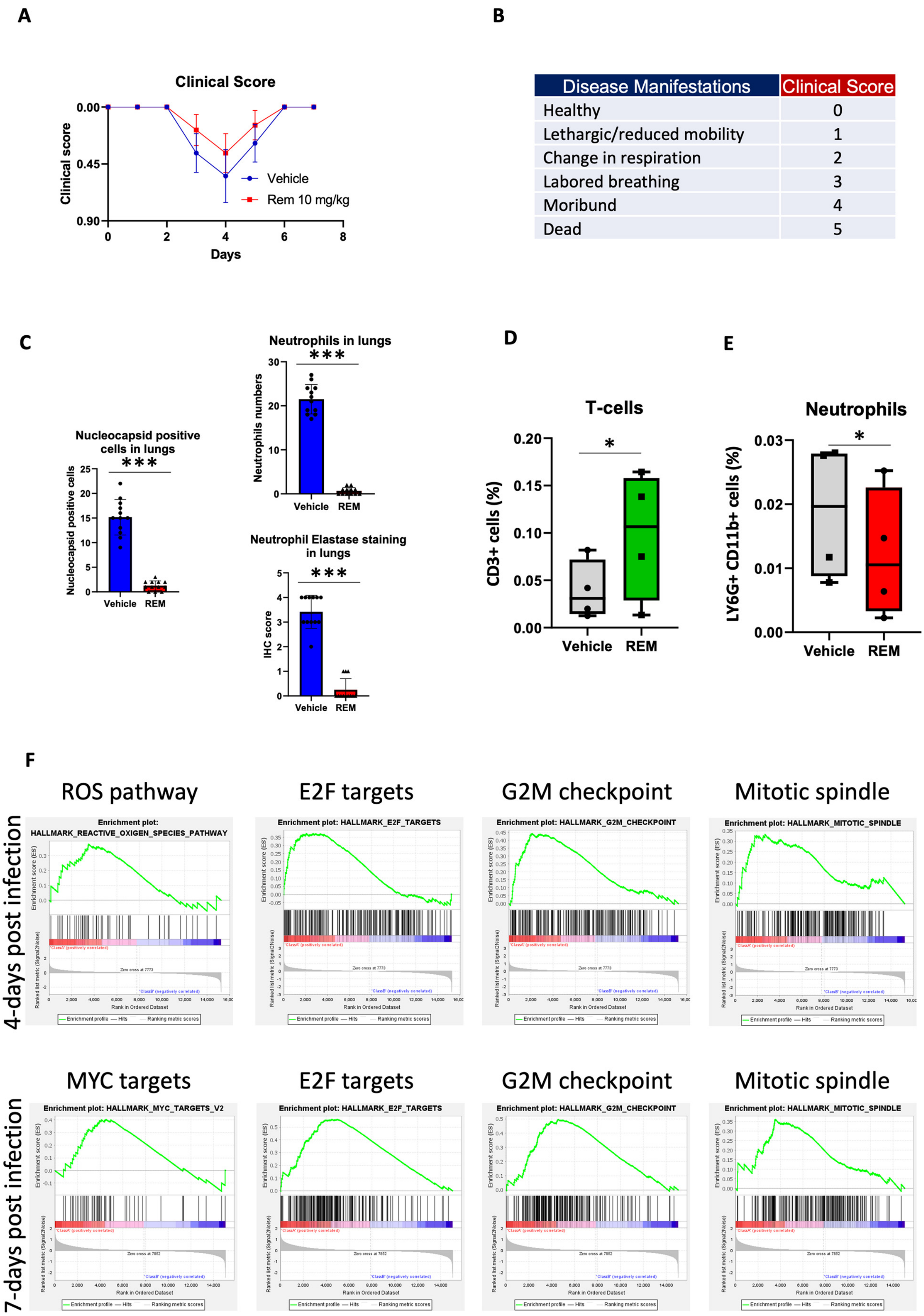
A
Clinical Score
Clinical score
0.00
0.45
0.90
Days
Vehicle
Rem 10 mg/kg
B
Disease Manifestations | Clinical Score
Healthy | 0
Lethargic/reduced mobility | 1
Change in respiration | 2
Labored breathing | 3
Moribund | 4
Dead | 5
C
Nucleocapsid positive cells in lungs
Nucleocapsid positive cells
Neutrophils in lungs
Neutrophils numbers
Neutrophil Elastase staining in lungs
IHC score
Vehicle
REM
***
D
T-cells
CD3+ cells (%)
*
E
Neutrophils
LY6G+ CD11b+ cells (%)
F
4-days post infection
ROS pathway
E2F targets
G2M checkpoint
Mitotic spindle
Enrichment plot: HALLMARK_REACTIVE_OXIGEN_SPECIES_PATHWAY
Enrichment plot: HALLMARK_E2F_TARGETS
Enrichment plot: HALLMARK_G2M_CHECKPOINT
Enrichment plot: HALLMARK_MITOTIC_SPINDLE
7-days post infection
MYC targets
Enrichment plot: HALLMARK_MYC_TARGETS_V2
Enrichment score (ES)
Ranked list metric (Signal2Noise)
Rank in Ordered Dataset
Enrichment profile
Hits
Ranking metric scores

 © The Author(s)

Figure EV5. Clinical and immunological readouts in SARS-CoV-2-infected mice treated with remdesivir.

(**A**) Clinical score of SARS-CoV-2-infected mice treated with vehicle or 10 mg/kg REM over 7 days ($n = 8$ mice per group). (**B**) Table of disease manifestations and corresponding clinical score. (**C**) Quantification of SARS-CoV-2 nucleocapsid positive cells, neutrophils, and neutrophil elastase staining in IHC of lung sections from SARS-CoV-2-infected mice treated with REM vs. vehicle ($n = 12$ image fields per staining). (**D**, **E**) frequencies of (**D**) T-cells and (**E**) neutrophils in the lungs after treatment with REM vs. vehicle ($n = 4$ mice per group). The box plots show minima, maxima, mean, 75, and 25 percentiles. (**F**) GESA transcriptomic analysis of lung RNA from vehicle group vs. REM-treated group. (Class A: vehicle group. Class B: REM-treated group). Data were presented as mean ± SEM. Data information: "*n*" indicates biological replicates. Data were presented as mean ± SD. Statistical significance was calculated by one-way ANOVA with Bonferroni correction (**A**) or two-tailed unpaired *t*-test (**C**–**E**). $^{*}P \le 0.05$, $^{***}P \le 0.001$., ns non-significant.

© The Author(s)
